# Factors associated with child health service delivery by female community health volunteers in Nepal: findings from a national survey

**DOI:** 10.1186/s12913-020-05424-x

**Published:** 2020-06-19

**Authors:** Hari Krishna Bhattarai, Pratik Khanal, Vishnu Khanal, Kiran Regmi, Narendra Raj Paudel, Liladhar Dhakal, Samikshya Singh

**Affiliations:** 1 JSI Research and Training Institute Inc., Kathmandu, Nepal; 2 Nepal Development Society, Bharatpur, Nepal; 3 Institute of Medicine, Tribhuvan University, Kathmandu, Nepal; 4 Former Secretary of the Ministry of Health and Population, Principal Investigator of the FCHV survey 2014, Kathmandu, Nepal; 5 Tribhuvan University, Kathmandu, Nepal

**Keywords:** Child health service, Female community health volunteers, Nepal

## Abstract

**Background:**

Nepal has made a significant improvement in child survival in the last few decades and the involvement of female community health volunteers (FCHVs) has been crucial in such achievement. While there have been many studies on child health in Nepal however, rarely explored the status and factors associated with the child health service provided by these v***o***lunteers. This study aimed to identify the factors associated with the child health service delivery by FCHVs.

**Methods:**

A national survey was conducted in 2014 in Nepal that included 4302 FCHVs using the structured questionnaire across the 13 geopolitical domains of the country. Factors associated with the use of child health services was examined using Chi-square test (χ^2^) followed by logistic regression.

**Results:**

Overall, 62.6% of FCHVs provided at least one child health service. Those FCHVs who utilized money from the FCHV fund, conducted health mothers’ group meeting, involved in local committees and those who supported antenatal care and outreach clinics related activities had higher odds of providing child health services. Similarly, FCHVs equipped with the stock of Cotrimoxazole tablet, Zinc tablet, Oral Rehydration Salt packets were more likely to provide child health services. The province-wise analysis showed that FCHVs from Province 5 and Sudur Paschim Province were more likely to provide child health services compared to their counterparts from province 1. Technology-wise, FCHVs who were using mobile were more likely to provide child health services.

**Conclusions:**

FCHVs are important human resource in providing child health services in Nepal. To improve child health service delivery by FCHVs; availability of key commodities, involvement of FCHVs in regular health mothers’ group meeting, use of mobile phone, involvement in other public health programs and social networks, and utilization of the FCHV fund need to be taken into consideration.

## Background

Global under-five deaths have decreased from 12.6 million in 1990 to 5.4 million in 2017 [1]. After Sub-Saharan Africa, South East Asia has the highest burden of under-five deaths. Diarrhoea and pneumonia are still the major killer diseases among the under-5 year children globally and nationally [2, 3]. Acute Respiratory Infection (ARI) and associated fever, dehydration from diarrhoea and malnutrition are the important contributing causes of childhood morbidity and mortality in developing countries. Most child deaths are caused by diseases that are readily preventable or treatable with proven, cost-effective and quality-delivered interventions [2].

In developing countries, health system suffers from a chronic shortage of human resources. In this context, the mobilization of community health workers or volunteers has been seen as a cost-effective and promising health system intervention [4]. Nepal started the Female Community Health Volunteers (FCHVs) program in 1988. This program was designed to enhance Nepal’s primary health care service delivery network, improve community participation, and expand the outreach of health services in doorsteps. The program was established in all 75 districts of the country by 1995 [5].

Nepal initiated community-based diarrhoeal disease control program in 1982 involving local health workers to promote home-based oral rehydration therapy and case management at the health facilities. This intervention, which also involved FCHVs after their introduction in the health system, had an important role in reducing child morbidity and mortality attributed by diarrhoea [6]. In 1997, an evaluation of community-based pneumonia case management supported by the World Health Organization (WHO) further paved the way to involve the FCHVs to treat the pneumonia cases [7]. Given that the percentage of diarrheal cases treated with Oral Rehydration Salt (ORS) and zinc by FCHVs among the total diarrhoeal cases reported throughout the county was 64% in 2014/15 and 65% in 2015/16, they are an important cadre to curb child deaths and sickness [3]. Recognizing the importance of community-based interventions, the Government of Nepal initiated community based-integrated management of childhood illness program (CB-IMCI) in 1999 to address major childhood killer diseases (Diarrhoea, ARI, Measles, Malaria and Malnutrition). This intervention involved FCHVs to recognize and treat pneumonia and diarrhoea in children under 5 years of age, and refer the sick neonates and young infants with any danger signs including malnutrition [6].

The role of FCHVs was crucial to achieving Millennium Development Goal 4 targets (reduction of child mortality by two-thirds in 2015 as compared to 1990) in Nepal [8, 9] which was also exemplary to the rest of the world. However, evidence regarding the FCHV program in Nepal [10–12] has shown that the FCHV program has been inconsistent in delivering high-quality community health services across the nation. Given their contribution to bridging the gap between health facility and community, it is necessary to motivate and capacitate this workforce for providing community health services including child health. To do so, it is important to understand the factors that are affecting FCHVs performance. However, very few studies have explored the factors affecting their performance. Previous studies done in small settings [10, 11] suggest that local leadership, work burden, geographical difficulties, inadequate training, limited monitoring and evaluation and incentive issues affect the service delivery by FCHVs. In 2014, the Ministry of Health conducted a national survey on FCHVs [13] for assessing the performance of the FCHV program. In this context, this study aimed to identify child health services provided by FCHVs and determine the factors associated with it using the data from the national survey. The findings of this study will serve as evidence for the policymakers and program managers to focus on management aspects of FCHVs to improve the child health status of the country.

## Methods

### Study design and sampling population

Data for the study was obtained from a nationally representative FCHV Survey conducted in 2014 [13]. The survey was a cross-sectional study based on cluster design. For the study purpose, the country was divided into 13 domains considering ecological and developmental regions of the country. These 13 domains included 36,050 wards. Wards were the primary sampling unit. In each domain, the sampling frame of wards was created and wards were selected randomly to get sample size per domain. In total 257 urban and 4045 rural wards were selected for the sample. The details of the sampling procedure have been reported in the survey report [10].

The study included 4302 FCHVs. They were interviewed using structured questionnaires which can be accessed in survey report [13]. These questionnaires were finalized based on the feedback received through pretest and experts. Trained enumerators were mobilized with a window-based tablet to collect the data. Information related to service delivery was obtained through review of the FCHV service register. A mobile platform namely “Enketo” linked with SurveyCTO was used to collect and analyze data. Detailed data collection methods have been discussed in the survey report [10].

### Variables

#### Outcome variable

The outcome variable in this study was the child health service provided by the FCHVs. It was defined as any one of the five services recorded in the service register of the FCHVs. These five services included: distribution of ORS packets to diarrhoea cases, distribution of Zinc tablet to a child suffering from diarrhoea, examine the child for ARI, distribution of cotrimoxazole pediatric tablet to a child suffering from pneumonia, and counselling or referral service to the malnourished child.

#### Independent variables

The independent variables included characteristics related to socio-demography, work profile of FCHVs, supports received by FCHVs, governance of the FCHV program, availability of commodities and service delivery by FCHVs.

Socio-demographic variables included the age, caste, educational status, residence and province. In the survey, FCHVs were labelled as “literate” if they had an education level of sixth grade and above, or if those with less than that level of schooling but could fully or partially read a simple sentence written in the card. The work profile of the FCHVs included time taken to reach the health facility, use of mobile phone and years of work experience. The time taken to reach health facility was grouped in three categories (less than 30 min, 30–60 min and more than 1 h) and years of work experience was also grouped into three categories (less than 1 year, 1–5 years and more than 5 years). Support received by FCHVs included information about whether they have received basic training (yes/no), dress allowance (yes/no), incentive received (yes/no) and use of FCHV fund (yes/no). Information on the availability of key commodities included the availability of ORS packets (yes/no) and zinc tablet (yes/no) to treat diarrhoea and Cotrimoxazole tablet (yes/no) to treat pneumonia. Service delivery by the FCHVs included information on the involvement of FCHVs in antenatal care (ANC) services: counseling to pregnant women, involved in testing pregnancy or providing misoprostol for preventing postpartum haemorrhage in case of home delivery (yes/no), conducted health mothers’ group meeting (yes/no), supported in immunization clinics (yes/no) and supported in conduction of Primary Health Care-Out Reach Clinics (PHC-ORC) (yes/no). Governance part of the FCHV program included information about affiliation with FCHVs right based organization (yes/no) and involvement with other local committees (yes/no).

### Statistical analyses

Descriptive analysis was performed and presented as frequencies and percentages. Then, the bivariate analysis (chi-square test) was performed to examine the association between dependent and independent variables. The multi-collinearity analysis was performed and variables which had correlation value less than + 0.5 were considered in the regression analysis. Multiple logistic regressions were employed to determine which variables could best explain the child health service delivery by FCHVs. Adjusted Odds Ratios (AOR) were presented with their 95% confidence intervals (CI) and *p*-value of less than 0.05 was considered as statistical significance. Sampling weight was used to adjust the sampling distribution using “svy” command in Stata 13 (StataCorp LP, College Station, TX, USA) [14].

### Ethics

The ethical approval for this study was obtained from the Nepal Health Research Council (Reference no: 122/2014) which is the government body that approves human researches and monitors such studies within the country. Written informed consent was taken from the study participants before the data collection and personal identifiers were removed during the data analysis.

## Results

### Services provided by FCHVs

Overall, 62.6% of the FCHVs had provided at least one child health service within the last 3 months before the survey. Among the five child health services, 51.6% of the FCHVs had treated at least one case of diarrhoea with ORS in the last 3 months, which was the highest among all the services provided and 9.3% had counselled or referred the malnourished child which was the lowest among all the services provided (Table 1).

**Table 1:** Proportion of FCHVs who provided different child health services (*n* = 4302)

**Child health services**^**a**^	**N(%)**
Diarrhoea treated with ORS	2220 (51.6)
Diarrhoea treated with Zinc	1910 (44.4)
Examined for a cough or cold	1906 (44.3)
Treated with cotrimoxazole for possible pneumonia cases	1045 (24.3)
Malnourished children counselled or referred	400 (9.3)
Provided at least one child health-related services	2693 (62.6)

^a^Multiple responses

### Relationship of child health service delivery with the socio-demographic profile of FCHVs

In the bivariate analysis, child health service delivery by FCHVs was associated with their literacy status, ethnic group, residence and province. FCHV’s age was not associated with child health service delivery (Table 2).

**Table 2:** Association between socio-demographic characteristics and delivery of child health services (*n* = 4302)

**Background characteristics**	**Total** **n (%)**	**Provided child health services** **% (95% CI)**	***p*value**
**Age**			
< 25 yr	204 (4.38)	58.04 (52.11, 63.76)	0.477
25–39 yr	1735 (39.09)	63.24 (60.15, 66.23)	
40–54 yr	1869 (43.62)	62.84 (59.43, 66.12)	
55+ yr	494 (12.9)	61.79 (57.96, 65.48)	
**Literacy**			
Illiterate	708 (18.4)	52.44 (47.67, 57.16)	0.001*
Literate	3592 (81.6)	64.92 (62.56, 67.22)	
**Caste**			
Hill caste	1812 (39.71)	66.95 (64.1, 69.68)	0.008*
Terai caste	512 (14.08)	58.17 (50.12, 65.82)	
Dalits	260 (6.35)	68.19 (60.94, 74.64)	
Janajatis	1628 (37.69)	59.49 (56.73, 62.19)	
Others	90 (2.17)	51.73 (35.72, 67.4)	
**Residence**			
Urban	257 (0.8)	55.66 (51, 60.21)	0.008*
Rural	4045 (99.2)	62.71 (60.26, 65.09)	
**Province**			
1	825 (17.53)	62.36 (58.05, 66.49)	0.001*
2	521 (19.36)	57.35 (51.84, 62.69)	
Bagmati	702 (16.76)	57.97 (55.19, 60.7)	
Gandaki	361 (12.75)	55.84 (50.75, 60.81)	
5	778 (14.85)	73.12 (70.11, 75.93)	
Karnali	410 (9.13)	59.87 (54.63, 64.89)	
Sudurpaschim	705 (9.63)	77.48 (73.39, 81.1)	
**Years of work experience**			
< one year	131 (3.04)	51.4 (40.86,61.32)	< 0.05
1–5 year	733 (17.04)	65.24 (61.45,68.84)	
> 5 years	3438 (79.92)	62.56 (59.95,65.09)	

*P* value: from Chi-square test

### Relationship of child health service delivery with other background characteristics

In the bivariate analysis, child health service delivery was associated with the use of mobile phone, time to reach the nearest public health facility, training received, dress allowance received, availability of Zinc, availability of ORS, availability of Cotrimoxazole, support in ANC service, support in PHC-ORC clinic, conduction of health mother’s group meeting, use of FCHV fund and involvement in local committees. On the other hand, child health service delivery was not associated with the incentive received, support in immunization clinic and FCHVs’ involvement in a right based organization (Table 3).

**Table 3:** Association between other background characteristics and child health service delivery (*n* = 4302)

**Characteristics**	**Total** **n (%)**	**Provided child health services% (95% CI)**	***p*value**
**Use of mobile phone**			
No	727 (17.38)	49.30 (43.34,55.27)	< 0.001*
Yes	3575 (82.62)	65.46 (63.31,67.54)	
**Time to reach the nearest public health facility**			
< 30 min	1462 (34.36)	59.84 (56.25,63.32)	0.018*
30–60 min	1645 (38.1)	64.90 (61.77,67.9)	
> 60 min	1195 (27.54)	63.05 (60.81,65.24)	
**Received basic training**			
No	195 (3.79)	54.09 (47.99,60.07)	0.009*
Yes	4107 (96.21)	62.99 (60.42,65.48)	
**Received dress allowance**			
No	193 (4.5)	52.61 (46.39,58.76)	0.008*
Yes	4109 (95.5)	63.12 (60.45,65.71)	
**Received monetary/ non-monetary incentive other than dress allowance**			
No	2735 (64.18)	61.5 (58.24,64.66)	0.068
Yes	1567 (35.82)	64.71 (62.37,66.97)	
**ORS in stock**			
No	986 (24.69)	52.15 (47.52,56.74)	< 0.001*
Yes	3315 (75.31)	66.1 (63.9,68.24)	
**Zinc in stock**			
No	1993 (46.62)	54.93 (52.44,57.4)	< 0.001*
Yes	2308 (53.38)	69.41 (66.74,71.95)	
**Cotrimoxazole pediatric in stock**			
No	2224 (51.47)	56.2 (53.59,58.78)	< 0.001*
Yes	2077 (48.53)	69.5 (66.7,72.16)	
**Supported in ANC services in the last 3 months**			
No	346 (8.65)	39.02 (32.43,46.03)	< 0.001*
Yes	3956 (91.35)	64.89 (62.23,67.45)	
**Provided support in PHC-ORC in the last 3 months**			
No	2260 (51.58)	58.07 (55.43,60.66)	< 0.001*
Yes	2042 (48.42)	67.53 (65.19,69.78)	
**Supported in immunization clinic in the last 3 months**			
No	1753 (40.25)	58.34 (55.96,60.68)	0.913
Yes	2549 (59.75)	65.55 (62.76,68.25)	
**Conducted health mother’s group meetings in the last 3 months**			
No	239 (4.74)	37.93 (30.79,45.64)	< 0.001*
Yes	4063 (95.26)	63.88 (61.11,66.56)	
**Used FCHV fund**			
No	1743 (39.96)	58.22 (54.64,61.71)	< 0.001*
Yes	2399 (60.04)	66.38 (63.7,68.97)	
**Involved in local committees**			
No	1596 (38.68)	57.26 (53.91,60.54)	< 0.001*
Yes	2706 (61.32)	66.05 (63.85,68.18)	
**Associated with FCHVs right based organization**			
No	3821 (89.98)	61.6 (58.92,64.2)	0.491
Yes	481 (10.02)	72.12 66.62,77.02)	

*P* value: from Chi-square test

### Factors associated with child health service delivery

The results of multiple regression analyses are presented in Table 4. The FCHVs from Muslim and other tarai caste were less likely to provide child health services compared to the hill caste FCHVs (AOR = 0.52, 95% CI: 0.32, 0.84). Likewise, FCHVs from Province 5 (AOR = 1.83, 95% CI: 1.43, 2.32) and Sudurpaschim province (AOR = 2.45, 95% CI: 1.89, 3.17) were twice more likely to provide child health services than the FCHVs from Province 1.

**Table 4:** Determinants of child health service delivery by FCHVs

Characteristics	Adjusted Odds Ratio (95% CI)
**Caste**	
Hill caste	Ref
Terai caste	0.92 (0.70, 1.22)
Dalits	1.28 (0.93, 1.77)
Janajatis	0.92 (0.78, 1.09)
Muslim and tarai others	0.52 (0.32, 0.84)**
**Province**	
1	Ref
2	1.27 (0.95, 1.69)
Bagmati	0.94 (0.75, 1.18)
Gandaki	0.93 (0.70, 1.22)
5	1.83 (1.43, 2.32)**
Karnali	0.90 (0.68, 1.19)
Sudurpaschim	2.45 (1.89, 3.17)**
**ORS in stock**	
No	Ref
Yes	1.44 (1.22, 1.70)**
**Zinc in stock**	
No	Ref
Yes	1.48 (1.27, 1.72)**
**Cotrimoxazole Pediatric tablet in stock**	
No	Ref
Yes	1.27 (1.09, 1.48)*
**Provided support in the ANC clinics in last 3 months**	
No	Ref
Yes	2.22 (1.72, 2.87)**
**Provided support in PHC-ORC in the last 3 months**	
No	Ref
Yes	1.34 (1.15, 1.55)**
**Involved in mother’s group meeting**	
No	Ref
Yes	1.66 (1.21, 2.27)**
**Used FCHV fund**	
No	Ref
Yes	1.31 (1.14, 1.51)**
**Use of mobile phone**	
No	Ref
Yes	1.64 (1.35, 1.98)**
**Involved in local committees**	
No	Ref
Yes	1.29 (1.11, 1.49)**

** = *p* < 0.01, * = *p* < 0.05; Adjusted full model

Availability of health commodities was associated with the FCHV’s service performance. FCHVs having ORS packets (AOR = 1.44, 95% CI: 1.22, 1.70), Zinc tablets (AOR = 1.48, 95% CI: 1.27, 1.72) and Cotrimoxazole tablets (AOR = 1.27, 95% CI: 1.09, 1.48) in stock were more likely to provide child health services. The FCHVs who supported pregnant women in ANC related activities (AOR = 2.22, 95% CI: 1.72, 2.87), supported PHC-ORC (AOR = 1.34, 95% CI: 1.15, 1.55) and conducted health mothers’ group meeting (AOR = 1.66, 95% CI: 1.21, 2.2) within the last 3 months had significantly higher odds of providing child health services than those who did not. The FCHVs who utilized money within last 1 year from the FCHV fund (AOR = 1.31, 95% CI: 1.14, 1.51) were more likely to provide child health services compared to those who did not use the money from the fund. Similarly, FCHVs who used a mobile phone (AOR = 1.64, 95% CI: 1.35, 1.98) had higher odds to provide child health services as compared to those who did not use the mobile phone. Likewise, FCHVs who were involved in different local committees (AOR = 1.29, 95% CI: 1.11, 1.49). were more likely to provide the child health services than those who were not involved in such committees.

In the adjusted analysis, literacy, residence, time to reach the nearest health facility, training received and dress allowance received were not significantly associated with the child health service delivery provided by FCHVs (Table not shown).

## Discussion

The sharp decline in child mortality in Nepal after the 1980s has been attributed to inexpensive community-based interventions [7, 15–17] led by local health workers and FCHVs. Studies around the world have shown that community-based interventions with minimal training to community health volunteers and ensuring the availability of life-saving key commodities contributes to the decline in child mortality [18–21]. Our study findings demonstrate that nearly two out of three FCHVs were involved in providing at least one child health service in the last 3 months before the survey. There is no doubt about the importance of FCHVs in rendering child health services. For instance, 67% of all the diarrhoea cases in the country were reported by FCHVs in fiscal year 2017/18 [22]. However, it is also relevant to understand the reasons for not providing child health services by the remaining FCHVs. This might be related to motivational factors as well as supply-side factors, partly explained by our study findings, but need further exploration.

In our study, availability of key commodities (Cotrimoxazole tablet, Zinc tablet and ORS) with FCHVs was significantly associated with providing child health services. The limited availability of commodities severely restricts FCHVs’ ability to provide services and affects the community trust on them. The major reason for this is due to the inadequate stock of drugs in health facilities [23] which is again the consequence of procurement and transportation hurdles. Reducing commodity stock-out rates across health facilities in Nepal and equipping FCHVs with these commodities could thus reasonably be assumed to contribute to improved service delivery and child health outcomes. Though the importance of local health workers cannot be ignored, mobilization of FCHVs has supported in bringing services closer to the community in a country which has been suffering from a chronic shortage of human resources for health. A study from Nepal [12] has indicated that women did not prefer to contact FCHVs during the illness of their child because of their incompetency and lack of medicines. Thus, for an effective FCHV program, their mobilization needs to be continuously monitored and supervised by local health workers with regular competency-based training and sufficient supply of logistics.

The Government of Nepal has created an FCHV fund- a micro-credit fund, which is managed by FCHVs. They use this fund in income-generating activities. Our study revealed that the utilization of money from the fund was positively associated with child health service delivery by FCHVs. The fund might have strengthened their economic status, increased the sense of belongingness and improved their performance which is also supported by a qualitative study done in Nepal [10].

The FCHVs who supported PHC-ORC and ANC related activities were more likely to provide child health services as compared to those who did not support these activities. In these activities, the FCHVs are involved in educating and counseling couples on family planning; distributing commodities; promotingANC, institutional delivery, and postnatal care for mother and newborn [22]. Involving FCHVs in these wide ranges of interventions would aid in the integration of maternal, child and newborn services thus leading to the continuum of care and better outcomes in child survival [21, 24]. It is however equally important to consider the work burden of FCHVs while task shifting and also capacitate FCHVs to deal with cultural and religious issues that surround during pregnancy and childbirth in Nepal as identified in other studies [25, 26].

Our study showed that the participation of FCHVs in health mother’s group meeting was associated with the delivery of child health services. These meetings are unique platforms to discuss different health issues and are attended by local women. Studies from Nepal have shown that frequent interactions between mothers and FCHVs were related to the use of child health services from FCHVs [12] and reduction in underweight and stunting status among children [27]. Similarly, a study from Makwanpur, Nepal [28] and Jharkhand and Orissa of India [29] had shown that participatory intervention involving women’s group can decrease both maternal and neonatal mortality and improve service utilization.

Our study findings showed that the use of the mobile phone by FCHVs was associated with child health service delivery indicating that a mobile phone could play a potential role to improve child health outcomes. The use of mobile phone and wireless technology has a huge potential to improve the health and wellbeing of the resource constraint communities through communication and exchange of skills among health workers and with communities [30, 31]. The use of the mobile application to improve maternal and neonatal health outcomes has been studied among FCHVs in Baglung and Dhanusha districts of Nepal which also has shown promising results [32–34]. This might be because FCHVs are likely to be contacted through mobile phone in case of emergency or other health needs by the communities leading to increased service utilization of child health services [33].

Our study findings demonstrated that FCHVs who were involved in the local level committees were more likely to provide child health services than those who were not involved in similar committees. RB Khatri, SR Mishra and V Khanal [35] however are of the view that the involvement of FCHVs in other non-health programs such as forest user groups, community development groups, education, and microcredit and saving groups could compromise their working hours in the health sector leading to poor performance. We put forward that such involvement may open opportunities for social networking leading to higher self-esteem and increased performance. However, monitoring of the activities of FCHVs needs to be done by health facilities and local governments for effective performance management of these cadres.

Our study findings indicate that incentives do not affect the delivery of child health services by FCHVs. This might be because of their volunteering role and that FCHVs are motivated by social recognition which is also supported by a qualitative study done in Nepal [36]. Studies have however shown that the issue of fair compensation for FCHVs needs to be addressed [10, 36, 37] as economic insecurity is a strong barrier to volunteering. Motivational activities like the provision of training and dress allowance for FCHVs were not significantly associated with the delivery of child health services in this study. The reason might be that FCHVs had joined volunteerism with the least expectation. In our study, education of FCHVs was not significantly associated with the child health service delivery. This finding contrasts with a study from Dhanusha, Nepal [38] where the educational status of FCHVs was associated with their knowledge and performance on maternal and child health services.

Nepal is in the early stage of implementation of federalism and the constitution has identified health as a fundamental right. While the federal government is responsible for overseeing broader policy, the province and local governments are responsible for the management of the health services [39, 40]. In the changed federal governance, the task of managing FCHVs comes under the direct responsibility of the local level governments (municipality, rural municipality) who were previously managed by local health posts and primary health centres. The role of local-level governments would thus be crucial to motivate these cadres for contributing to the health of the communities. The FCHVs are complimentary cadres to improve the health of the communities and their effort alone might not be sufficient. It is also necessary that there is increased community demand for health care and the availability of quality health services at health institutions.

This study is based on a large sample size and the findings can be generalizable to the entire country. However, there are inherent limitations to the study. Firstly, its cross-sectional study design does not allow establishing causation. Secondly, this study only focuses on the FCHV’s perspective and thus there is an indication of further research to understand the community and service provider’s side perspectives. Furthermore, this study doesn’t identify the main reasons for not providing or providing child health services. Future research may be needed to explore these factors in detail. Despite these limitations, this study provides useful evidence for the policymakers and program managers for understanding the factors influencing the performance of FCHVs in providing child health services. The evidence can be utilized for efficient and effective utilization of these cadres for improved child survival.

## Conclusions

This study revealed that availability of the essential commodities (Cotrimoxazole tablet, Zinc tablet and ORS), use of mobile phone, conduction of health mothers’ group meeting, involvement in local level committees and support in other health programs and utilization of FCHV fund were the determinants of child health service delivery by FCHVs in Nepal. Ensuring a sustainable means to supply these essential commodities and encouraging the involvement of FCHVs in community-based health services through adequate support system will help to improve child health status in Nepal. The existing FCHV program can benefit from this evidence by motivating these cadres for strengthening child health services at the community level.

## Data Availability

Availability of the data is available upon request to the corresponding author.

## Abbreviations

AOR: Adjusted Odds Ratio
ARI: Acute Respiratory Infection
CB-IMCI: Community Based Management of Childhood Illness
CI: Confidence Interval
FCHVs: Female Community Health Volunteers
ORS: Oral Rehydration Salt
PHC-ORC: Primary Health Care Out Reach Clinic
WHO: World Health Organization
